# Metformin improves ovarian follicle dynamics by reducing theca cell proliferation and CYP-17 expression in an androgenized rat model

**DOI:** 10.1186/s13048-018-0392-1

**Published:** 2018-03-01

**Authors:** Roberta Rassi Mahamed, Carla Cristina Maganhin, Gisela Rodrigues Silva Sasso, Manuel de Jesus Simões, Maria Candida Pinheiro Baracat, Edmund Chada Baracat, José Maria Soares-

**Affiliations:** 1 Department of Gynecology, Escola Paulista de Medicina, Universidade Federal de São Paulo, São Paulo, Brazil; 2 Laboratory of Molecular and Structural Gynecology (LIM 58) of Disciplina de Ginecologia, Departamento de Obstetrícia e Ginecologia, Hospital das Clínicas, Faculdade de Medicina da Universidade de São Paulo, Avenida Dr Arnaldo, 455- sala 2113, São Paulo, SP Zip Code 01246903 Brazil; 3 Department of Morphology and Genetics, Escola Paulista de Medicina, Universidade Federal de São Paulo, São Paulo, Brazil

**Keywords:** Metformin, Steroidogenesis, Ovary, Rat

## Abstract

**Background:**

Metformin influences insulin receptor signaling, which might interfere with the proliferation of ovarian follicular structures and steroidogenesis. We hypothesize that reductions in glucose and insulin levels might interfere with CYP-17 expression and histomorphological changes in an androgenized rat model. The aim of this study was to analyze the effect of metformin on CYP-17 expression, follicular dynamics, and proliferative parameters in neonatally androgenized female rats.

**Methods:**

Thirty-six newborn rats were randomly allocated to the following three groups on the third day of life: control (CG, *n* = 12), androgenized (GA, *n* = 12), and androgenized + metformin (GAmet, *n* = 12). The GA and GAmet animals were administered 0.1 mL of testosterone propionate (1.25 mg/animal) diluted in castor oil (vehicle) in a single dose; the CG rats received a subcutaneous injection of the vehicle in the dorsum. After 90 days, gavage treatment was initiated, distilled water was administered to the CG and GA rats, and metformin (150 mg/kg) was administered to the GAmet animals. The treatment was administered daily for six weeks. Following anesthesia, blood was drawn for biochemical measurements, and the ovaries were removed for histological and immunohistochemical analyses of Ki67, VEGFA and CYP17 expression. The glucose and insulin levels were also measured.

**Results:**

The comparison of the GA and GAmet animals revealed that metformin decreased the weight as well as the glucose and insulin levels, slowed the proliferation of the theca interna and interstitial cells, as evidenced by Ki-67 and VEGF-A expression, and diminished CYP17 expression in the analyzed ovarian structures. In addition, metformin reduced the number of degenerating follicles and interstitial cells and improved angiogenesis.

**Conclusion:**

Metformin improves the carbohydrate metabolism, reduces proliferation, and decreases CYP-17 expression in the follicular structures of androgenized rats.

## Background

Polycystic ovarian syndrome (PCOS) is a common endocrine disorder in women of reproductive age, but its etiology remains unclear. PCOS might have multiple causes, and one possible cause might be related to its association with hyperinsulinemia and insulin resistance [1]. The genesis and pathophysiology of these changes are known to be related to hyperandrogenism [2]. Hyperinsulinemic insulin resistance acts indirectly on oocyte competence and oocyte quality, increasing the production of ovarian androgens and decreasing the synthesis of hepatic sex hormone-binding globulin (SHBG) [3].

In PCOS, insulin sensitivity is thought to regulate the expression of glucose transporters in granulosa cells by reducing glucose uptake within the oocyte, thereby also reducing the resources available for energy metabolism [3].The insulin receptor sensitizer substance acts as an intracellular second messenger, regulating the activities of insulin and glucose metabolism and transport [4] and decreasing hyperandrogenism and insulin resistance in PCOS women [5].However, the exact mechanisms through which an increase in androgen levels leads to ovulation disorder are not yet fully understood. Therefore, the use of animal models is important to better understand the actions involved [6].

Several animal models, including nonhuman primates, sheep, and rodents, have been established to study PCOS. However, a whole-animal model that mimics all the features associated with human PCOS has not yet been established [6, 7].An injection of testosterone propionate, if administered during the first days of life in rats, leads to a state of permanent estrus and keeps the vagina from opening, which might indicate a reduction or absence of ovulation due to hormonal dysfunction [8].This method is used to assess the effects of androgens on follicular physiology [9] and changes in carbohydrate metabolism [1]. High testosterone doses increase the serine phosphorylation of insulin receptor substrate-1, inducing insulin resistance and compensatory hyperinsulinemia, which produce a metabolic disturbance [10].The effects of this method during the first days of life in an animal model are likely due to epigenetic modifications.

The ovarian CYP complexes are important for ovarian steroidogenesis. The CYP17a1 and CYP11a1 genes encode 17-α hydroxylase and 17,20-lyase, respectively, two key enzymes involved in the synthesis and metabolism of androgens as well as the cholesterol side-chain cleavage enzyme, P450 [10]. The end result is pregnenolone conversion into androgen, primarily in the theca interna [11].The induction of permanent estrus through insulin administration increases CYP17 expression and serum androgen levels in female rats and results in the breakdown of the estrous cycle [12]. In our previous work [8], we demonstrated that our PCOS rat model exhibited elevated insulin levels, which could explain the permanent cycle and the increase in androgen production [12]. However, there are scarce data regarding the ability of metformin to reverse this effect because previous studies primarily demonstrated the effect of the drug on weight and the glucose/insulin ratio [8].

In rats, metformin improves ovarian function in animals with permanent androgen-induced estrus [13–15]. In our previous studies, the percentage of neonatally androgenized rats with PCOS was approximately 50 and 10% become pregnant after metformin treatment. The reason that fertility was restored in such a small number of animals might be related to the disruption of ovarian activity or to endometrial abnormalities. Therefore, our study aimed to assess the effects of metformin on the follicular dynamics, CYP-17 expression and proliferation parameters in neonatally androgenized female rats.

## Methods

Virgin adult female albino EPM-1-Wistar rats (*Rattus norvegicus albinus*) aged 90 to 120 days from the Center for the Development of Experimental Models (CEDEME, UNIFESP-EPM), Federal University of São Paulo – Paulista School of Medicine (UNIFESP-EPM), were used in this study and handled according to ethical principles for experiments. This project was approved by the Institutional Review Board of UNIFESP-EPM (No. 0351/11).

The animals were transported to and housed in the animal laboratory of the Division of Histology and Structural Biology, Morphology Department, UNIFESP-EPM, where they were confined to plastic cages with metal grids. The animals were administered standard rations (Labina-Purina) and water ad libitum and were maintained at a room temperature of 21 °C to 25°Cand a 12-h light/dark cycle (lights on from 7 a.m. to 7 p.m.) with artificial lighting from fluorescent lamps (40-watt daylight model).

After acclimatization for approximately 15 days, the animals were grouped for mating: two female rats for each male rat. Each group was placed and housed in a cage from 7 p.m. to 7 a.m. without water or food. The following morning, the mating test developed by Hamilton and Wolfe [16], which is based on the presence or absence of sperm in the vagina of the female rats, was performed. If positive, the test day was regarded as the beginning of pregnancy (first day).

Following parturition, newborn rats were separated by sex on the third day of life according to the anogenital distance [17]. Thirty-six newborn female rats were selected and housed together with their mothers, but the male newborns were euthanized. On the third day of life, 24 female newborns were administered 0.1 mL of testosterone propionate diluted in castor oil (vehicle) (1.25 mg/animal) via the dorsal subcutaneous route by inserting the needle from the dorsal region to the cervical region [8, 18]. The remaining 12 newborn female rats constituted the control group and received only vehicle.

All female newborns were housed together with their mothers while nursing (30 days) under the environmental conditions described above. After this period of nursing, the animals were separated into groups of four rats per cage. After 90 days, a vaginal smear was collected from each animal on four consecutive days. The testosterone propionate-treated animals exhibited a closed vagina, and their vaginal smear test demonstrated that they were in permanent estrus [18, 19].

The animals were allocated to the following groups: control group (CG), 12 female rats that received no medication and only 0.5 mL of distilled water by oral gavage for six consecutive weeks; androgenized group (GA), 12 female rats in permanent estrus that were treated with testosterone propionate and 0.5 mL of distilled water by oral gavage for six consecutive weeks; and androgenized + metformin group (GAmet), 12 female rats in permanent estrus that were administered testosterone propionate followed by metformin by oral gavage for six consecutive weeks. A total of 36 animals were used in this study.

All GAmet animals were weighed before and after the metformin treatment. The medication, 150 mg/kg of metformin [7] prepared in diluted water, was introduced into the stomach of each animal by gavage with a gastric catheter [20]. The dose of metformin was determined based on previous studies [8, 20].Vaginal smears were collected from the GAmet animals [21] daily for six weeks, starting on the first day of metformin use, to characterize the estrous cycle. Afterward, the animals in the control group were sacrificed during the proestrus phase, and the GAmet animals were sacrificed at the time of vaginal opening and proestrus phase. The other animals were sacrificed at any time after the treatment because it was impossible to collect vaginal smears.

Prior to sacrifice, the female rats were fasted for 12 h and then intraperitoneally anesthetized with 15 mg/kg Xylazine (Rompun®; Bayer, Brazil) and 30 mg/kg Ketamine (Ketalar®; Pfizer, Brazil). Each animal was immobilized on a cork board. The thoracic wall was then opened with a xiphopubic incision of the wall for intracardiac collection of blood for the insulin and fasting glucose measurements and removal of both ovaries. The ovaries were immersed in 10% formaldehyde for fixation and subsequent histologic processing using the methodology described by Michalany [22].

### Insulin and glucose measurements

The fasting glucose levels were measured using the hexokinase enzymatic method, and the insulin levels were measured using the Coat-A-Count (Diagnostic Products Corporation, LA, USA) method according to the manufacturer’s instructions. The test was performed using a solid-phase radioimmunoassay with iodine 1251 for quantitative measurement of the serum insulin levels. The inter-assay precision was approximately 10.2% (coefficient of variation), and the intra-assay precision was approximately 6.5% (coefficient of variation). The assay sensitivity was 4.2 micro IU/mL [23].

### Glucose/insulin ratio calculation

The glucose/insulin ratio was calculated using the following formula: fasting glucose (ng/mL)/fasting insulin (ng/mL) X 10 [7].

For the calculations, the unit of glucose was converted from mg/dL to ng/mL.

### Morphological analysis

The ovaries from each animal were fixed in 10% buffered formaldehyde for 24 h, dehydrated in increasing concentrations of ethyl alcohol, cleared in xylol, and embedded in liquid paraffin in a 60 °C oven using the methodology described by Michalany [22]. Subsequently, the blocks were cut into 4-μm sections with a Leica microtome. The sections were placed on slides and incubated in a 37 °C oven for 24 h. The slides were then stained with hematoxylin-eosin and mounted in Entellan for histomorphometric and immunohistochemical analyses.

### Histomorphometric analysis

Histomorphometry was conducted using a computer coupled to a light microscope (Carl Zeiss, Weesp, The Netherlands) and a high-resolution camera (AxioCam MRC, Carl Zeiss). The Vision REL 4.6 (Carl Zeiss) image analyzer program was used [24, 25]. Measurements were collected using the Image lab-Softium™ software program (São Paulo, Brazil).

Ten measurements from each slide were collected for each animal. The ovaries were included, and sections were cut from the central region to the outer boundary. The structures of interest were measured in microns. Histological quantification encompassed the following items: (1) thickness of the surface epithelium (μm); (2) number of antral follicles (No./10 fields); (3) number of non-antral follicles (No./10 fields); (4) number of degenerating follicles (No./10 fields); (5) number of corpora lutea (No./10 fields); and (6) number of interstitial cells (No./area of 780 μm^2^).

The follicles were counted and classified according to previously established criteria [26–28]. Two independent histologists who were blinded to the groups counted the follicles.

The ovarian follicles were divided into three categories: non-antral, antral, and degenerating. The corpora lutea were also counted.

Non-antral follicles were defined by the absence of the antral cavity and characterized as primordial, primary, secondary, or tertiary follicles [29]. The primordial follicle exhibited a layer of squamous cells surrounding the oocyte. The primary follicle exhibited an oocyte surrounded by a layer of cuboidal granulosa cells. The secondary follicle exhibited two to eight layers of granulosa cells and no antral cavity surrounding the oocyte. Tertiary follicles displayed more than eight layers of granulosa cells and lacked an antral cavity [27].

The follicles with an antral cavity, regardless of their size, were considered antral follicles. The degenerating follicles exhibited pyknotic nuclei in the granulosa layer, according to the categorization reported by Pedersen and Peters [26]. The corpus lutea exhibited characteristic luteal cells with a voluminous nucleus and vessels [30]. Two observers who were blinded to the groups separately collected the histomorphometric measurements.

### Immunohistochemical analysis of the ovaries

The histological tissue sections were fixed in buffered 10% formaldehyde for 24 h and then embedded in paraffin. Sections with a thickness of 3 μm were cut on a Minot-like microtome and placed on slides that had previously been treated with 5% silane. The sections were subsequently incubated overnight in a solution with the following antibodies: (1) anti-Ki67(1:200; Spring Bioscience Corp.; [SP6]; M3060); (2) anti-VEGFA (1:200; Spring Bioscience Corp.; [SP28]; M3281), and (3) anti-CYP17A1 (1:200; Santa Cruz Biotechnology; [C-17]; SC-46081). The sections were then incubated with a secondary antibody and a Dako kit (Dako LSAB + Sys, Peroxidase Universal K0690–1). The sections were then washed and incubated with a freshly prepared streptavidin-biotin immunoperoxidase solution (LSAB Dako kit) according to the manufacturer’s instructions. After washing, the bound enzyme was visualized after an additional incubation with the enzyme in the presence of 1% 3,3′-diaminobenzidine (Dako K3468–1). The negative control consisted of incubation with non-specific goat antibodies at the same concentration as the primary antibody for each immunohistochemical reaction (Ki67, VEGF and CYP17A1). All the sections were also stained with hematoxylin.

Using the protocol reported by Panzan et al. [31], the results were categorized by applying a scoring system based on the intensity of the reactions and the frequency of stained cells. The presence of a brownish color in the tissue was adopted as a positive reaction (i.e., binding of the antigen to the primary antibody). Immunoexpression was evaluated at 400× magnification using the semiquantitative method.

The percentages of stained epithelial cells were graded as follows: 0% (grade 0), 1 to 25% (grade 1), 26 to 50% (grade 2), 51 to 75% (grade 3), and 76 to 100% (grade 4) of the cells in a representative area. The intensity of the immunoreactions was scored as negative (0), mild (1), moderate (2), or strong/intense (3). The sections with no staining were deemed negative. The total index was calculated using the following formula: Total index = percentage of immunopositive cells x immunoreaction intensity. The results are expressed as scores ranging from 0 to 12.

The expression of Ki67 antigen was determined as a percentage (%) of positively stained cells. In all cases, at least 500 cells in a 100-μm^2^ area were counted per animal. The investigators were blinded to the group allocation.

### Statistical analysis

The data are expressed as the means ± standard deviations (M ± SD). The statistical analyses were performed with GraphPad Prism 5.0® (GraphPad Software Inc.) software. The distribution of the data was analyzed using the Kolmogorov-Smirnov test. If the distribution was normal, the data were analyzed by one-way ANOVA followed by Tukey’s test, a procedure for analyzing variables with multiple comparisons. However, the Kruskal-Wallis test and the Dunn post hoc test were employed to analyze the immunohistochemical data, which did not exhibit a normal distribution. Additionally, the correlations of the glucose/insulin ratio and weight with the CYP17 data of the glucose level with the weight were assessed using Spearman rank correlation coefficients. Statistical significance was attained if the *p*-value was less than 0.05 (α < 5%).

## Results

**Fig. 1:**
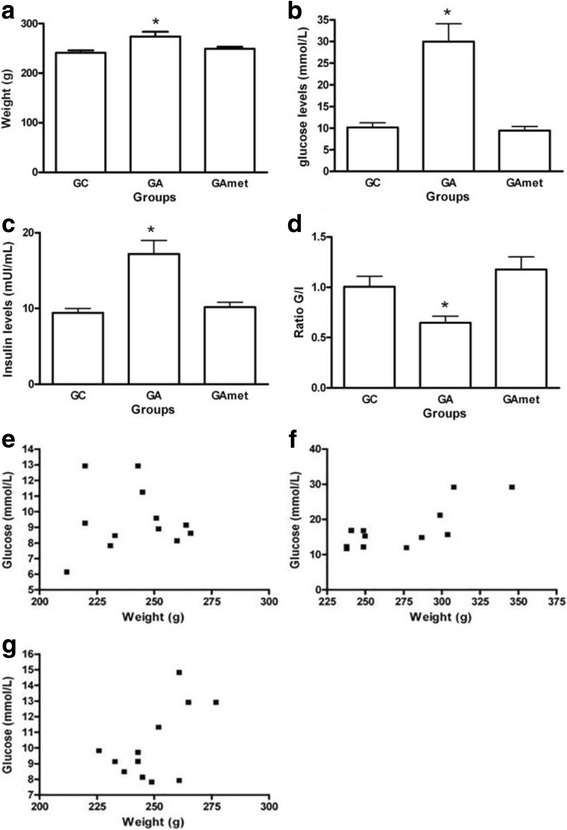
Weight measurements and biochemical analysis. **a** Weight of the rats in the following groups: CG (control group), GA (androgen group), and GAmet (metformin group). *p* = 0.049 (ANOVA and Tukey’s test). **b** GA rats exhibited the highest glucose values, *p = 0.038 (Kruskal-Wallis and Dunn tests). **c** The insulin levels of the GA group were higher than those of the other groups, *p* < 0.01(Kruskal-Wallis and Dunn tests). **d** The lowest glucose/insulin ratio was observed in the GA group, * *p* < 0.01. **e** Spearman rank test of the correlation of glucose with weight in CG rats (no significant correlation). **f** Spearman rank test of the correlation of glucose with weight in GA rats (no significant correlation). **g** Spearman rank test of the correlation of glucose with weight in GAmet rats (no significant correlation)

The fasting glucose levels, insulin levels, glucose/insulin ratio, animals’ weights, and Spearman correlation between glucose levels and weight are presented in Fig. 1.

The GA rats presented higher glucose and insulin levels and increased weight compared with the rats in the CG and GAmet groups (*p* < 0.05) (Fig. 1a, b, and c). Additionally, compared with the other groups, the GA rats exhibited the lowest G/I ratio (*p* < 0.01) (Fig. 1d). No correlation between the weight data and glucose levels was identified using the Spearman test (Fig. 1e, f, g).

The histomorphometric data for the ovaries (Table 1) showed that the GA rats have greater quantities of degenerating follicles and interstitial cells than the CG or GAmet rats (*p* < 0.01). No significant difference was observed between the non-antral and antral follicles or in the thickness of the ovarian surface epithelium among the three study groups. Nevertheless, an absence of corpora lutea was observed in the GA rats, and the GAmet rats had fewer corpora lutea than the CG rats (*p* < 0.01, Table 1).

**Table 1:** Ovary histomorphometry and VEGFA, Ki67 and CYP17 immunohistochemical data of the study groups

Variable	CG (*n* = 12)	GA (*n* = 12)	GAmet (*n* = 12)
Degenerating follicles (No./slide)	4.11 ± 1.07	5.87 ± 1.87*	3.10 ± 1.10
Corpora lutea (No./10 fields)	8.26 ± 2.81**	–	3.06 ± 1.32
Non-antral follicles (No./10 fields)	3.23 ± 1.41	5.98 ± 3.88	3.77 ± 2.80
Antral follicles (No./fields)	1.97 ± 1.87	2.89 ± 1.89	2.98 ± 1.95
Interstitial cells (No./780-μm^2^ area)	162.29 ± 23.21	313.18 ± 26.38*	172.45 ± 37.85
Epithelial thickness (μm)	8.28 ± 0.10	8.59 ± 0.19	8.30 ± 0.15
VEGFA (index)			
Granulosa cells	2.6 ± 0.2	9.6 ± 0.3*	4.2 ± 0.6
Theca interna cells	3.8 ± 0.2	10.7 ± 0.6*	5.1 ± 0.2
Interstitial cells	2.6 ± 1.6	9.3 ± 0.3*	4.2 ± 0.2
Ki67 (%)			
Granulosa cells	32.0 ± 4.4	14.0 ± 3.3*	28.1 ± 2.5
Theca interna cells	16.0 ± 2.2	34.0 ± 1.5*	18.0 ± 3.1
Interstitial cells	1.2 ± 0.1	8.0 ± 0.3*	1.1 ± 0.4
CYP17 (%)			
Granulosa cells	1.1 ± 0.8	1.5 ± 1.1	1.2 ± 0.9
Theca interna cells	5.5 ± 1.7	9.3 ± 1.2*	6.2 ± 0.7
Interstitial cells	5.2 ± 0.8	7.4 ± 1.4*	4.2 ± 1.6

*Abbreviations*: *CG* control group, *GA* androgenized group, *GAmet* androgenized + metformin group. The data for all the groups are presented as the means±standard deviations. The histological results were analyzed using analysis of variance (ANOVA) and Tukey’s test. The VEGFA, Ki67 and CYP17 immunohistochemical data were analyzed using the Kruskal-Wallis test and the Dunn test. *p < 0.01 compared with the other groups and **p < 0.05 compared with the GAmet group

**Fig. 2:**
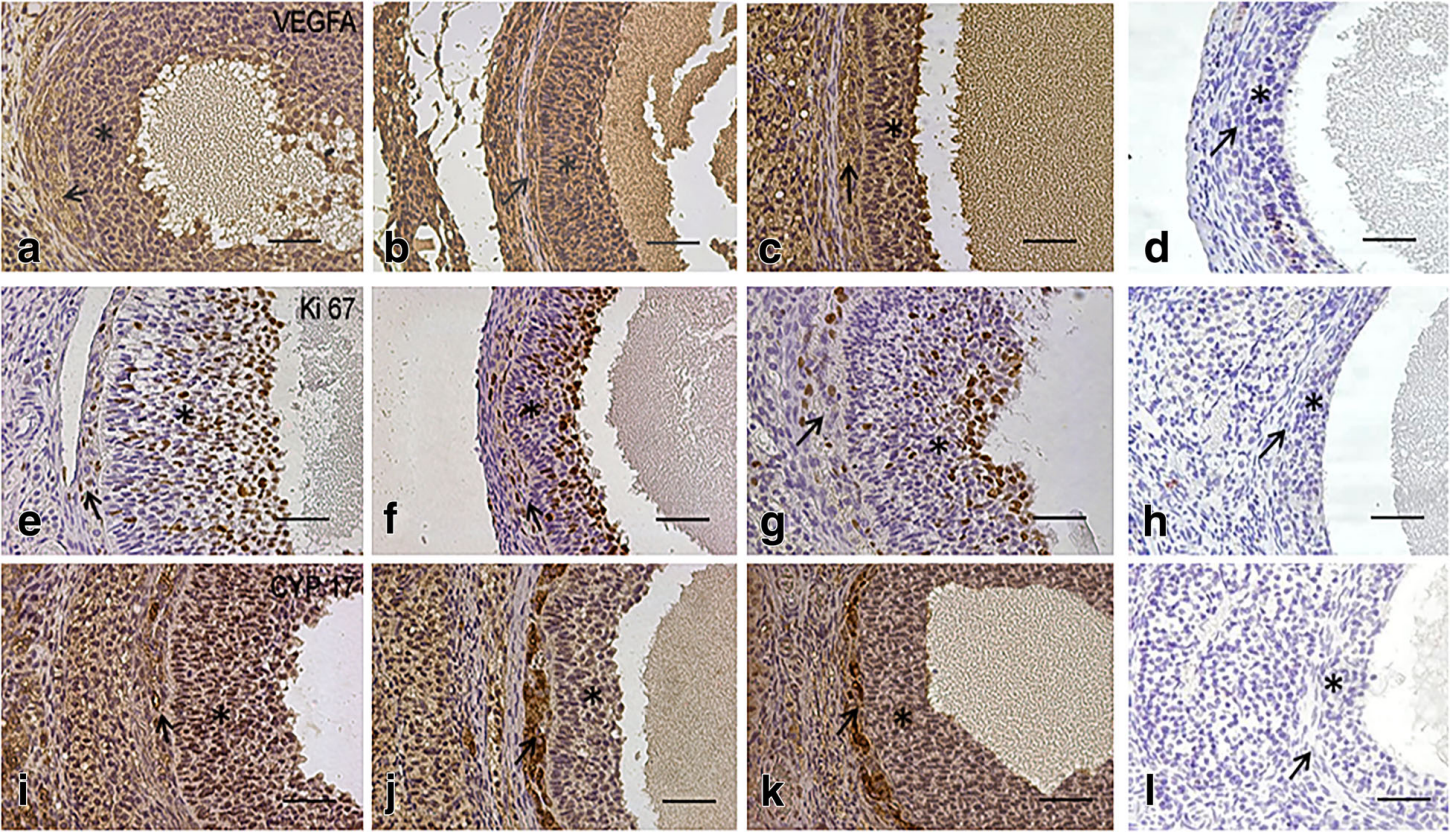
Photomicrographs of ovary sections immunostained for VEGFA (**a**, **b**, **c**); note the higher level of staining in the inner theca and granulosa cells in the GA group (**b**). Ki67 (**e**, **f**, **g**) staining was highest in the inner theca of the GA group (**f**) and lowest in the granulosa cells of the control group (**e**). CYP17 (**i**, **j**, **k**) expression was highest in the inner theca of the GA (**j**) group and in the granulosa cells of the control group (**i**). **d**, **h**, and **l** are negative controls for VEGFA, Ki67 and CYP17, respectively. Arrows: inner theca; asterisks: granulosa cells. Bars = 20 mm; magnification × 400

As shown through Ki67, VEGFA and CYP17 immunohistochemistry analyses (Table 1 and Fig. 2), the expression of Ki67 was increased in the granulosa cells and decreased in the theca interna cells and interstitial cells of the GAmet animals compared with the GA animals (*p* < 0.05). Compared with the CG animals, the expression of Ki67 in the GAmet animals was similar in the three structures under consideration.

VEGFA expression was reduced in the three structures of interest in the GAmet animals compared with the GA animals (*p* < 0.05). CYP17 was expressed at the lowest levels in the granulosa cells from the GA rats (*p* < 0.05) and at the highest levels in the theca interna and interstitial cells from the GA rats (*p* < 0.05, Table 1 and Fig. 2).

**Fig. 3:**
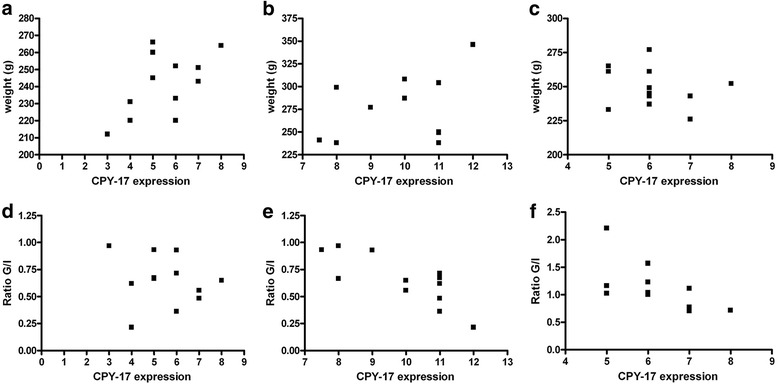
Graphical representations of the Spearman rank test for the correlation of the weight data withCYP17 expression in thecae cells of the (**a**) CG group (control group, no correlation), (**b**) GA group (androgen group, no correlation), and (C) GAmet group (metformin group, no correlation). **d**–f show graphical representations of the correlations between the glucose/insulin ratio data and CYP17 expression in theca cells. **d** CG, no correlation, (**e**) GA, significant correlation (*r* = − 0.81, *p* = 0.001), and (**f**)GAmet, significant correlation (*r* = − 0.61, *p* = 0.03)

Graphical representations of the correlation between the weight data and CYP17 expression in thecae cells and between the glucose/insulin ratio and CYP17 expression in theca cells are presented in Fig. 3. No correlation was observed between the weight data and CYP17 expression in any of the groups or analyzed structures (granulosa, theca and interstitial cells). We observed significant correlations between the expression in theca interna cells and the glucose/insulin ratio in the GA rats (*r* = − 0.81, *p* = 0.001) and the GAmet rats (*r* = − 0.61, *p* = 0.03). No correlation was observed between the granulosa or interstitial cells and CYP17 expression in the control group.

## Discussion

The effects of metformin on insulin metabolism have been previously evaluated [32]. However, the effects of this drug on the reproductive system, particularly the ovaries, is not well understood [3]. Here, we found that metformin partially restored the follicular dynamics with an increase in the number of luteal bodies and intense decreases in androgen-induced proliferation of thecae cells and CYP-17 expression, which is responsible for steroidogenesis and the maintenance of androgen production [12, 18, 33]. These effects restore the normal morphology of ovaries [34–37], but the aim of our study was not to observe the effect of metformin on the atresia of follicles. Further study is necessary to test this hypothesis.

In the study conducted by Shah and Patel [20], the androgenized rats exhibited a significant increase in CYP 17A1 expression in ovarian theca cells compared with the normal group, and the insulin levels were increased significantly. Treatment with metformin produced a significant decrease in the insulin levels [19, 38]. The results from another animal model using dehydroepiandrosterone to induce conditional hyperandrogenic PCOS demonstrated that treatment with metformin restored the percentage of endothelial and periendothelial cells and the level of ovarian VEGF [33]. Metformin also normalized the insulin concentration in PCOS rats and improved follicular development [33, 39]. These results suggest that metformin regulates vascular formation and stability in developing ovarian follicles from DHEA-treated rats. Metformin reduced the higher VEGF ovarian concentration. However, the researchers did not correlate the glucose/insulin ratio with the expression of CYP-17. In our study, we found that decreased CYP-17 was correlated with a low glucose/insulin ratio, which might reflect the insulin resistance detected in our animal model.

Hyperinsulinemia and hyperandrogenism are closely related [40, 41]. In fact, increased insulin levels are an important factor for the maintenance of hyperandrogenism. Together with luteinizing hormone (LH), insulin directly affects androgen production by increasing the androgen levels in theca interna cells in the ovaries in animal models [8, 12, 20]. Hence, the histological and immunohistochemical changes observed in our study might be associated with insulin metabolism, particularly in relation to CYP17 activity in theca interna cells. Metformin might reverse the effects of androgens on neonatal female rats through this mechanism and by modulating the insulin levels [20].

The mechanism of action of metformin involves AMPK in the insulin receptor signaling pathway [3]. Cell culture experiments have demonstrated that metformin directly inhibits rat ovarian theca-interstitial cell proliferation through AMPK [40, 42]. This action might explain the decrease in KI-67 and VEGF levels in theca cells obtained after metformin treatment in our animals. Metformin might indirectly decrease the production of liver growth factors, which might act on the ovaries [34, 41]. In our model, the number of IGF-1 receptors is decreased in granulosa cells and increased in theca interna cells, thus contributing to an increase in hyperandrogenism [35, 43]. Additionally, at high non-physiological concentrations, insulin binds to IGF-1 receptors, but if these receptors are blocked or reduced in number, insulin binds to the IGF-1 receptors on theca cells, thereby activating the receptors, which then increase androgen production [35]. Consequently, a reduction in insulin decreases CYP-17 expression and increases androgen production. Androgens, in turn, also lead to insulin resistance, particularly when administered to female rats during the prenatal period [36]. This fact might explain the higher glucose/insulin ratio values observed in the animals that were not treated with metformin in this study and that were previously reported [8].

VEGFA is an important agent involved in follicular growth and maturation, ovulation, and the development and maintenance of the corpus luteum [39]. Excess VEGFA levels might be related to ovarian hyperstimulation and the formation of multiple ovarian cysts. In our study, VEGF expression was altered following metformin administration to androgenized animals. In fact, metformin affects the regulation of angiogenesis and prevents excessive vascularization related to VEGF action, as demonstrated in the study conducted by di Pietro et al. [33]. In their study, female rats were androgenized with dehydroepiandrosterone (DHEA) and then treated with metformin, and the results were similar to those obtained in our model. VEGF is also sensitive to the insulin levels. Thus, the decreased insulin levels might justify the lower VEGF levels observed in the ovary [20, 44, 45].

Metformin is important in both ameliorating the glucose/insulin ratio in androgenized animals and improving ovarian follicular dynamics and enzymatic activity (CYP-17). Other insulin sensitizers, such as inositol, which re-establishes hormonal and metabolic parameters to the homeostatic levels, have been recently introduced and may have similar effect to metformin [46, 47].

The data reported in this manuscript were obtained from animal models and are not applicable to daily medical practice. This is a limitation of this study. Another limitation is that we did not evaluate liver function, notably the determination of IGF-1 and its binding proteins. Thus, there is a need for additional studies to verify the effects observed in this study, and further in vitro studies are needed to investigate the mechanisms through which metformin exerts its effects.

## Conclusions

The effects of metformin on follicular cells and on CYP-17 expression in testosterone-androgenized female rats included improvements in the glucose levels and changes to proliferation and ovarian steroidogenesis in androgenized rats.

## Abbreviations

CG: Control group
CYP11a1: Cytochrome P450 family 11 subfamily A member 1
CYP17a1: Cytochrome P450 family 17 subfamily A member 1
GA: Androgenized group
GAmet: Androgenized group with metformin
Ki67: Marker of proliferation Ki-67
PCOS: Polycystic ovarian syndrome
VEGF: Vascular endothelial growth factor A
